# Frequency and Antimicrobial Susceptibility Patterns of Canine and Feline Urinary Tract Pathogens: A 6-Year (2018–2023) Retrospective Study in Phoenix, Arizona, United States

**DOI:** 10.3390/microorganisms14081655

**Published:** 2026-07-29

**Authors:** Eliana De Luca, Sam Katzif, Kimberly J. Bussey, Catherine Cruz, Ogi Okwumabua

**Affiliations:** 1 College of Veterinary Medicine, Louisiana State University, Baton Rouge, LA 70803, USA; 2 College of Graduate Studies, Midwestern University, Glendale, AZ 85308, USA; 3 College of Veterinary Medicine, Midwestern University, Glendale, AZ 85308, USA

**Keywords:** antibiotic resistance, Arizona (USA), bacteria, companion animals, urinary tract infection, multidrug resistance, surveillance

## Abstract

This retrospective study is intended to contribute to the ongoing investigations of antimicrobial resistance in companion animals by specifically examining the demographics, sex, rates of resistance, and multidrug resistance of bacteria isolated from urine samples of cats and dogs suspected of urinary tract infections (UTIs) in Arizona, USA, between 2018 and 2023. *Escherichia coli* was the most isolated bacterium with an overall frequency of 43.4%, followed by *Enterococcus faecalis* (11.5%), *Proteus mirabilis* (10.7%), *Staphylococcus pseudintermedius* (9.9%), *Enterococcus faecium* (6.9%), and *Klebsiella pneumoniae* (6.3%). Each year, culture-positive canine urine samples were more frequent than positive feline samples, with summer months producing the majority of suspected UTI samples for dogs and cats. Age played a role for culture-positive urine samples only in female dogs, with no association between age or sex and the number of positive feline samples. Feline *Escherichia coli* and *Klebsiella pneumoniae* isolates demonstrated more resistance to penicillins + beta-lactamase inhibitors than canines. The feline *Enterococcus faecalis* isolates also revealed significantly more resistance than canine isolates to penicillins + beta-lactamase inhibitors. In terms of multidrug resistance, a significant difference was found between the isolates of both *Klebsiella pneumoniae* and *Proteus mirabilis* and other bacterial species. These findings contribute to the relevant role antimicrobial surveillance plays in the prevention and control of UTI pathogens in companion animals. Because dogs and cats often are in close contact with their owners, they may be potential sources for human infection and a One Health concern.

## 1. Introduction

Since their discovery in the early 20th century, antibiotics have represented a powerful therapeutic strategy for bacterial infections. The use of antibiotics has contributed to a global decrease in human morbidity and mortality rates as well as to increased life expectancy [1,2]. Antibiotics have been used not only for the prevention and treatment of diseases for both humans and animals but also to promote growth and enhance feed efficiency in animal agriculture and less commonly, in crop plants [3]. Between 2016 and 2023 antibiotic consumption in humans increased by 16.3% and is predicted to increase by 52.3% by 2030 [4]. In livestock, the increase from 2019 to 2030 is projected at ~18.6% [5].

As a result of this increasing use of antibiotics, there is a rising alarm in antimicrobial resistance (AMR), defined as the ability of a microorganism to resist the effects of antibiotic agents that were once effective in treating infections caused by these organisms [6]. This may be due to an intrinsic resistance, such as the constant presence of lipopolysaccharide (LPS) of Gram-negative bacteria, induced resistance, which may arise after exposure through the expression of multidrug efflux pumps, or acquired resistance resulting from mutations or the transfer of genetic material between bacteria via various mobile genetic elements, including plasmids, bacteriophages, and transposons [7,8].

Additionally, resistant bacteria can be transmitted between humans and animals through direct or indirect contact. This has been observed between dogs and their owners through the possible spread of fecal *Escherichia coli* with similar extended spectrum beta-lactamase genotypes, the transmission of *E. coli* phylotypes associated with UTIs, and the sharing of AMR of clinical importance [9,10,11,12]. Both of these mechanisms, AMR and contact, result in a serious threat to both public and animal health [13].

Several factors that have contributed to the growth of AMR include the overuse of common antimicrobials, excessive and improper use of antimicrobial drugs in both humans and veterinary medicine, and prior use of antimicrobials leading to an increased risk for infections by a resistant organism [8,14].

A growing concern is represented by the emergence of multidrug resistant (MDR) bacteria. MDR is defined as nonsusceptibility to ≥1 agent in ≥3 antimicrobial classes [15]. These MDR bacteria pose a serious challenge to the health and well-being of both humans and domesticated and wild animals. [16,17,18,19].

Various countries worldwide, such as France, the United Kingdom, Sweden, China, and the United States, have implemented surveillance programs to monitor the frequency and evolution of antibiotic resistance in commensal and zoonotic bacteria [20,21,22]. However, these efforts were mostly focused on food-producing animals, with subsequent limited data on the AMR of bacteria isolated from pets [23]. The lack of information on the overall AMR situation in companion animals poses serious challenges in light of their growing population, as well as their close contact with people, other animals, and the environment [23]. Furthermore, surveillance efforts of companion animals can provide data that may be clinically useful for the treatment of diseases in pets while reducing the antibiotic resistance risks caused by an improper use of drugs [15,24].

Bacterial urinary tract infections (UTIs) are one of the common infections diagnosed in dogs and cats, and antimicrobial therapy is indicated in most cases, greatly contributing to the development of antimicrobial resistance worldwide [25,26,27]. The most isolated bacteria from the dog urine sample are *Escherichia* spp., *Proteus* spp., *Staphylococcus* spp., and *Enterococcus* spp., while *Escherichia* spp., *Enterococcus* spp., and *Staphylococcus* spp. represent most of the isolates from cat samples [18].

To increase the available data on AMR bacteria isolated from companion animals, we provide an overview of feline and canine urine samples with suspected UTIs identified from 2018–2023 in Arizona. For this retrospective study, we combined the results of bacterial cultures with the demographics from potential cases to determine the occurrence of positive urine samples in cats and dogs according to age, sex, and seasonality, the frequency of isolated bacterial species, rates of antimicrobial resistance, and the number of MDR isolates for the 6 years of sample collection.

## 2. Materials and Methods

### 2.1. Inclusion Criteria and Data Source

Between January 2018 and December 2023, a total of 717 urine samples from canine and feline patients with a presumptive diagnosis of UTI were submitted for microbiology culture and antimicrobial susceptibility testing to the Microbiology Laboratory of the College of Veterinary Medicine at Midwestern University. Each of these patient samples was treated as an independent observation.

Since cystocentesis represents the diagnostic approach of choice for bacterial UTIs to reduce contamination and increase the accuracy of the data obtained, only samples collected by cystocentesis were included in this study [28]. Information concerning the age, sex, and season of collection was recorded from the submission form accompanying each specimen. For each bacterium isolated from these samples, an archived comprehensive report of antimicrobial susceptibility test results was downloaded from Sensititre^TM^ SWIN^TM^ Epidemiology Software v1.4 (Thermo Fisher Scientific, Waltham, MA, USA) to determine the antibiotic resistance pattern of each isolate. These data for pathogen occurrence, numbers of susceptible and resistant isolates, and related interpretation based on Clinical and Laboratory Standards Institute (CLSI) guidelines were recorded from each included year of sample collection. Through the use of the Thermo Scientific^TM^ Sensititre^TM^ Complete Automated AST System (Thermo Fisher Scientific, Waltham, MA, USA), control ranges, breakpoints, and interpretive categories for antimicrobial agents were automatically determined using the built-in interpretation criteria in the system that follows the CLSI standards for each isolate and year of analysis [29,30]. Because of the retrospective nature of the study, data on previous antimicrobial treatment, comorbidities, and other clinical notes for each patient were inconsistent and were not used in any analysis.

### 2.2. Culture, Bacterial Identification, and Antimicrobial Susceptibility Testing

All urine specimens were transferred to sterile containers and cultured within 2 h if held at room temperature (RT) or within 24 h when refrigerated at 4 °C. All urine samples were streaked using 0.01 mL calibrated loops onto two tryptic soy agar with 5% sheep blood plates, one MacConkey agar plate, and one Columbia nalidixic acid (CNA) plate (Thermo Fisher Scientific, Waltham, MA, USA). One tryptic soy agar with 5% sheep blood plate and the CNA plate were incubated with 5% CO_2_, while the second tryptic soy agar with 5% sheep blood and the MacConkey agar plates were incubated aerobically at 37 °C for 18 to 24 h until adequate growth was present. The approximate number of colony-forming units/milliliter (CFU/mL) of urine was determined by counting the resulting colonies on each plate and multiplying the colony number by the loop dilution factor (×100) for each specimen culture. Mixed cultures were initially distinguished by the presence of multiple colony morphologies on an original culture plate, and the CFU/mL was calculated as stated above.

For these cystocentesis-collected samples, isolates from both pure and mixed cultures that produced bacterial growth ≥1000 CFU/mL were treated equally as positive isolates and used for demographic, antimicrobial susceptibility, and multidrug resistance analyses. If no bacterial growth or <1000 CFU/mL was observed after 24 h of incubation, the isolate was recorded as not clinically significant and not included in any statistical analysis [15,31].

Identification of each of the bacterial isolates from pure or mixed cultures was based on bacterial morphology, Gram stain characteristics, and matrix-assisted laser desorption ionization time-of-flight mass spectrometry, MALDI-TOF MS (Bruker BioTyper^®^, Billerica, MA, USA) [32].

Antimicrobial susceptibility testing (AST) was carried out with the Thermo Scientific^TM^ Sensititre^TM^ Complete Automated AST System (Thermo Fisher Scientific, Waltham, MA, USA). Antimicrobial susceptibility testing was performed by the broth microdilution method using a commercially prepared, dehydrated 96-well microtiter minimum inhibitory concentration (MIC) panel for veterinary bacteria from companion animals. *Enterococcus faecalis* ATCC 29212, *Escherichia coli* ATCC 25922, *Pseudomonas aeruginosa* ATCC 27858, and *Staphylococcus aureus* ATCC 29213 were used as quality control strains following the manufacturer’s instructions and Clinical Laboratory Standards Institute (CLSI) guidelines [29]. The quality control strains and test bacteria were grown for 18 h on tryptic soy agar with 5% sheep blood and visually checked for purity before use. Colonies were picked and cells were standardized in Mueller-Hinton broth to a McFarland standard of 0.5 (1.5 × 10^8^ colony-forming units/mL) using the Sensititre^TM^ System nephelometer. Fifty microliters of the standardized bacterial suspension were dispensed into the wells of a 96-well microtiter plate containing different concentrations of test antimicrobials in either a Sensititre^TM^ companion animal Gram-positive (COMPGP1F) or a companion animal Gram-negative (COMPGN1F) plate (Thermo Fisher Scientific, Waltham, MA, United States). Plates were incubated aerobically for 18 h in a Sensititre AIRS HiQ^TM^ System (Thermo Fisher Scientific, Waltham, MA, USA). Results were interpreted as susceptible, intermediate, and resistant using the automated built-in MIC breakpoint values in the Sensititre^TM^ System specific for each year. All isolates that exhibited intermediate resistance were reclassified as susceptible in the statistical analysis for rates of resistance and multidrug resistance. Historically, the intermediate (I) category was ambiguous and did not effectively guide clinical decision-making. In practice, clinicians and related stakeholders often treated I as equivalent to resistance, grouping I and R together as “nonsusceptible.” This effectively created two resistant categories and only one clearly actionable susceptible category, limiting the usefulness of the classification. Reframing I alongside S recognizes that susceptibility exists on a spectrum and depends on achieving adequate drug exposure at the infection site. Thus, combining I with S produces a more clinically meaningful system with two levels of susceptibility and a single resistant category [33]. The antibiotic classes, agents, and concentration range for both the COMPGN1F and COMPGP1F 96-well plates can be found in Table 1.

The combination of antibiotic agents and antibiotic classes was used to identify multidrug-resistant (MDR) isolates [34]. Multidrug resistance was defined as nonsusceptibility to ≥1 agent in ≥3 antimicrobial classes, excluding drugs to which the investigated bacteria reported an intrinsic resistance [34]. Using the categories of very low (0.0–1%), low (>1–10%), moderate (>10–20%), high (>20–50%), very high (>50–70%), and extremely high (>70%), the rate of resistance to a specific antibiotic agent by a species of bacteria was calculated by the number of resistant samples (R) divided by the total number of tested samples minus samples with no results [35,36].

### 2.3. Descriptive Analyses

Categorical variables (age, sex, and seasonality) of each patient with a positive culture were evaluated from the years of collections (2018 to 2023). Age was presented in years, and animals were classified into 4 different age groups of young (0–2 years), mature (>2–6 years), senior (>6–11 years), and geriatric (>11 years) based on a classification scheme adapted from Garcês et al., 2022 [37]. There was no distinction made between intact and neutered males or intact and spayed females in the analysis of sex. Seasonality was divided into winter (late December–late February), spring (late February–late April), summer (May–September), and fall (October–mid-December) based on data presented by the National Weather Service for Arizona [38]. The occurrence of each pathogen over the years in both canine and feline bacterial isolates was investigated for percentage of total number, rates of resistance to antibiotic classes, and multidrug resistance.

### 2.4. Statistical Analyses

Statistical analysis was done in R (version 4.4.0). using the packages tidyverse (version 2.0.0), janitor (version 2.2.0), readxl (version 1.4.3), and stats (version 4.4.0) [39,40,41,42]. Additional statistical analysis was conducted using Prism 10 (GraphPad, Boston, MA). Relationships among seasonality, pet species, sex (regardless of reproductive status), and age were analyzed using a Poisson or negative binomial GLM and assessed for overdispersion. The model with the best fit was carried forward for post-hoc pairwise comparisons. Post-hoc pairwise comparisons of estimated marginal means were conducted using the emmeans package (version 2.0.3) in R. Estimated marginal means were computed from the fitted model, with all other covariates held at their observed means, and pairwise contrasts were expressed as ratios on the response scale following back-transformation from the log link. *p*-values were adjusted for multiple comparisons using the Tukey method.

Bacterial abundance across years and pet species was assessed in two ways. Relationships between proportions of bacterial species observed by year and/or pet species were assessed with Chi-square with Fisher’s exact test for post-hoc comparison. Relationships between year and pet species were evaluated within bacterial species using a Poisson or negative binomial GLM, followed by post-hoc pairwise comparisons as indicated above.

Antimicrobial class resistance was compared between cats and dogs using the Chi-square with Yates’ correction. Post-hoc evaluation of individual class differences in resistance proportions between dogs and cats was performed using the z-test of proportions and Benjamini-Hochberg adjustment for multiple comparisons. Graphical outputs used gglplot2 with code development assistance from OpenAI’s ChatGPT, using a customized R-focused GPT (“R Wizard”: ChatGPT, OpenAI, 22 May 2025, https://openai.com/chatgpt).

## 3. Results

### 3.1. Demographics of Feline and Canine Patients

Of our 717 samples, 57.9% (415/717) were culture-negative for bacterial growth, while 42.1% (302/717) were positive. The 717 samples were 73.1% (524/717) canine samples and 26.9% (193/717) feline samples. For the 302 positive samples, 84.8% (256/302) were collected from dogs and 15.2% (46/302) from cats. The number of positive samples in dogs was 48.9% (256/524) and in cats it was 23.8% (46/193). There was no increase in positive samples year over year in either cats or dogs. The incidence of positive samples was significantly different in dogs versus cats (Fisher’s exact test, *p* < 0.0001). Among urine samples submitted to this laboratory, canine samples were more frequently culture-positive than feline samples with an odds ratio of 2.930 (95% CI 2.025–4.274). No relationship was observed between the month of sample collection and the number of samples collected. However, the data suggested that binning months into seasonal bins might reveal a pattern. When using seasonal bins, suspected UTI samples collected in summer were more numerous than those collected in both spring (1.78 × more, *p* ≤ 0.01) and winter (1.61 × more, *p* ≤ 0.05) in both cats and dogs, while all other seasonal comparisons were not statistically significant.

Females made up 89.1% (41/46), and males contributed 10.9% (5/46) of the positive feline samples. For the positive canine samples, 81.2% (208/256) were collected from females and 18.8% (48/256) from males. Female dogs were more likely to test positive for bacterial pathogens compared to male dogs regardless of reproductive status (*p* ≤ 0.001; Poisson GLM with estimated marginal means correction).

Age played a role for female dogs but not male dogs, with Senior females (>6–11 years; 47.3%) having more positive urinary tract samples than any other age category (average 2.31 times, *p* ≤ 0.01; Poison GLM with estimated marginal means correction). Although the positive urinary tract samples, in cats were isolated from a population of 89.1% females with 43.5% (20/46) Senior patients, there was no association between age or sex and the number of positive feline samples.

### 3.2. Bacterial Species and Abundance

Within the 302 positive culture samples, 84.4% (255/302) produced pure cultures, while in 15.6% (47/302) of the samples more than one bacterial species was isolated. The total number of positive bacterial isolates for these 302 positive culture samples was 364, each with a CFU/mL of ≥1000 CFU/mL.

**Figure 1:**
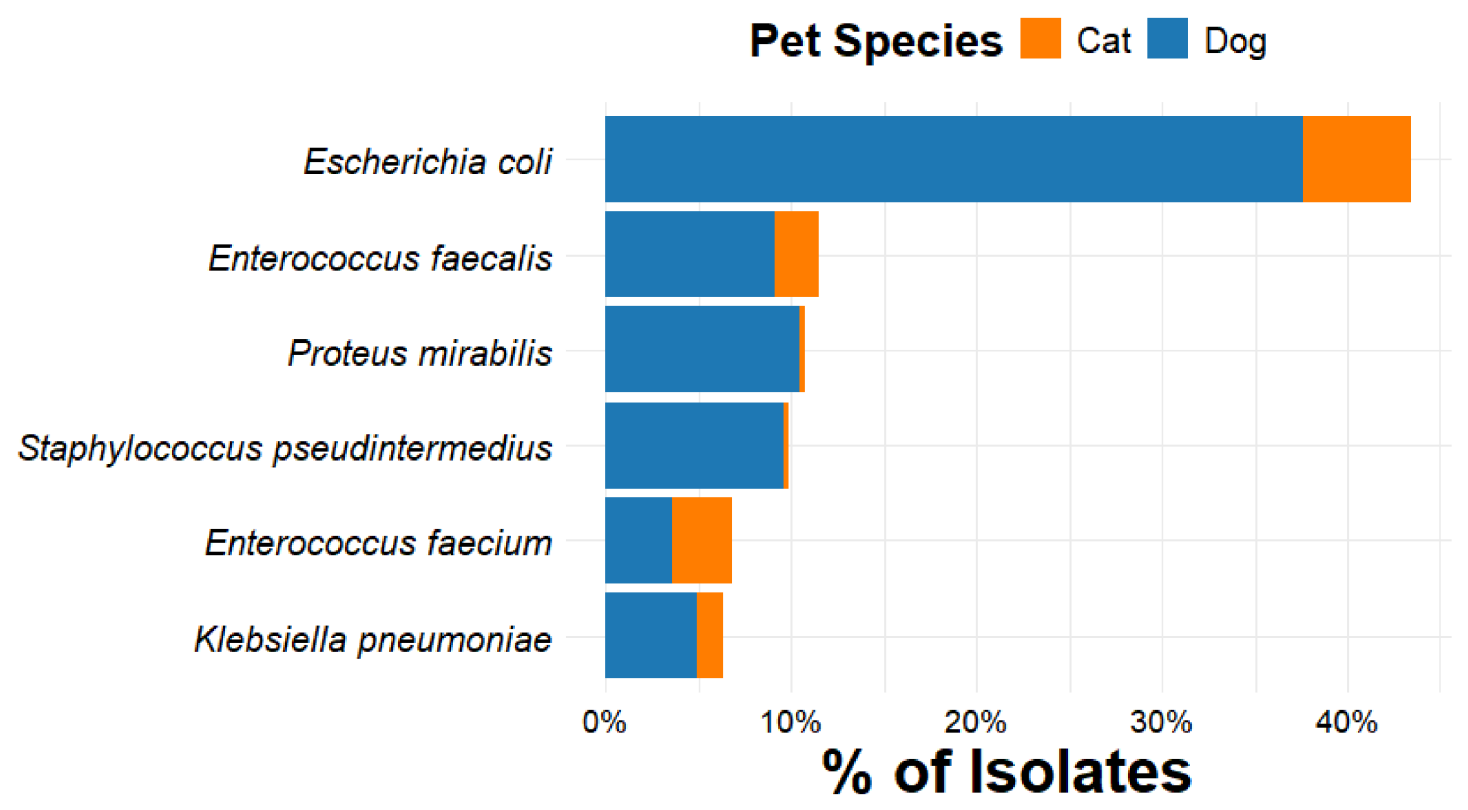
The six primary bacterial species isolated from cat and dog urine samples. Each of these six species of bacteria comprises ≥5% of the total number of bacteria isolated from cat and dog urine samples.

**Figure 2:**
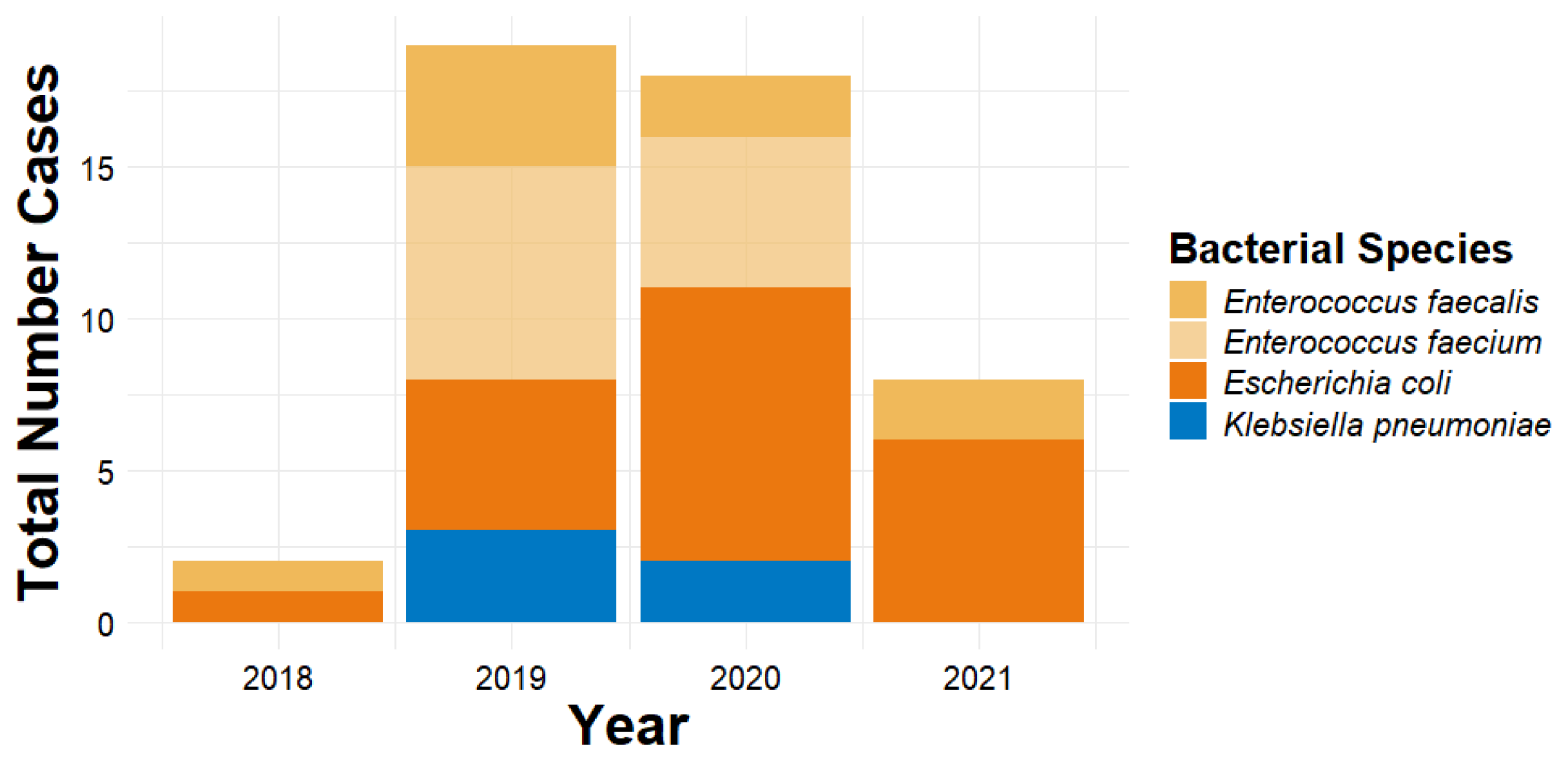
The four primary bacterial species isolated from cat urine samples. Each of these four species of bacteria comprises ≥5% of the total number of bacteria isolated from cat urine samples.

**Figure 3:**
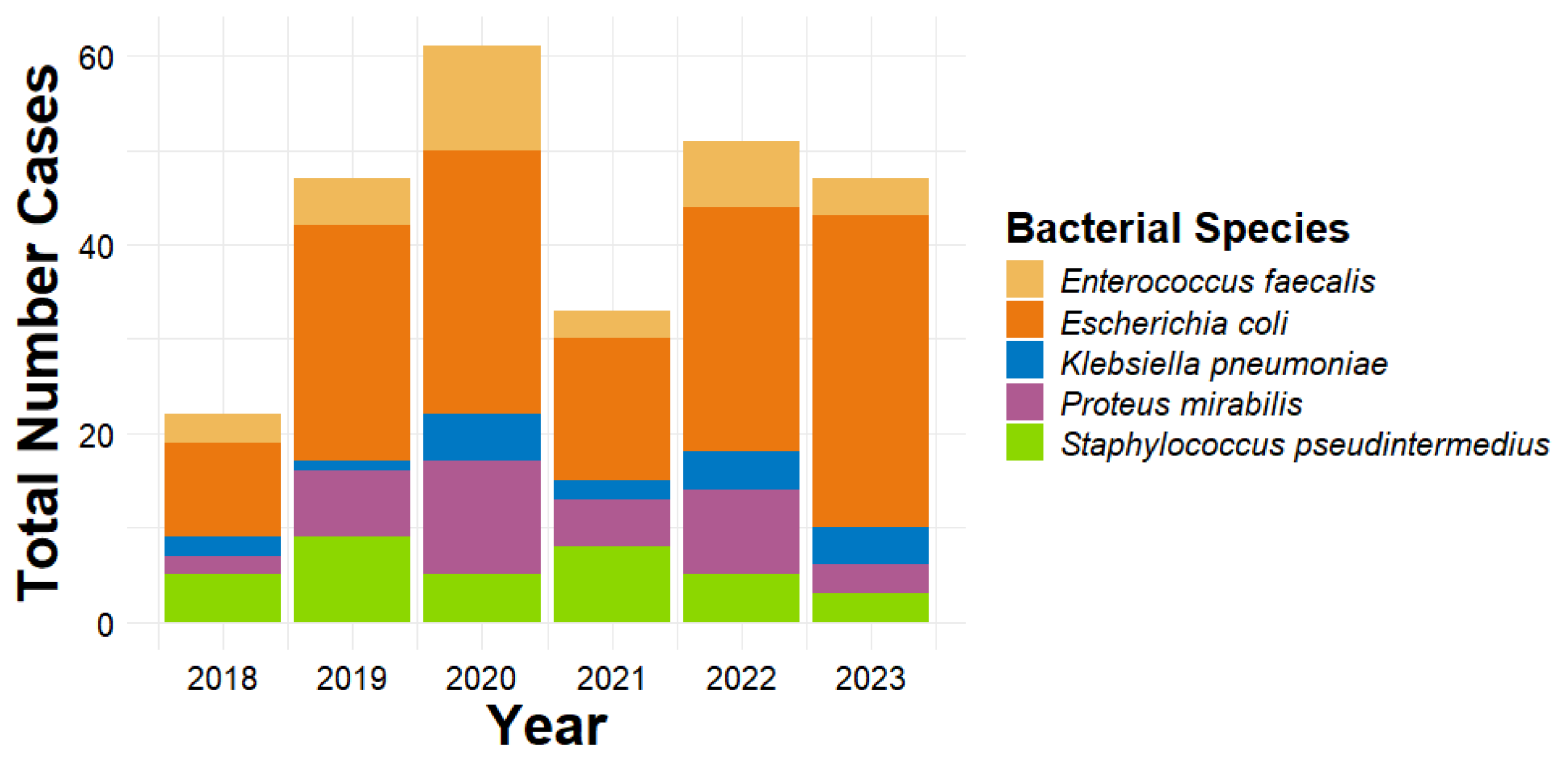
The five primary bacterial species isolated from dog urine samples. Each of these five species of bacteria comprises ≥5% of the total number of bacteria isolated from dog urine samples.

The total and yearly frequencies as well as numbers for each species of bacteria isolated in the six different years of collection for dogs and cats are reported in Table 2. There were 13 genera and 23 different species of bacteria identified from the 364 isolates cultured from urine samples of both dogs and cats. The frequencies for the top six species of bacteria (≥5% of the total number of bacterial isolates) identified from both dog and cat samples were *Escherichia coli* (43.4%, 158/364), *Enterococcus faecalis* (11.5%, 42/364), *Proteus mirabilis* (10.7%, 39/364), *Staphylococcus pseudintermedius* (9.9%, 36/364), *Enterococcus faecium* (6.9%, 25/364), and *Klebsiella pneumoniae* (6.3%, 23/364) (Figure 1). Separating dogs from cats, four bacterial species were isolated from feline urine samples at a frequency of ≥5%: *E. coli* (39.6%, 21/53), *E. faecalis* (17.0%, 9/53), *E. faecium* (22.6%, 12/53), and *K. pneumoniae* (9.4%, 5/53) (Figure 2). Five bacterial species were isolated from canine samples at a frequency of ≥5%: *E. coli* (44.1%, 137/311), *P. mirabilis* (12.2%, 38/311), *E. faecalis* (10.6%, 33/311), *S. pseudintermedius* (11.2%, 35/311), and *K. pneumoniae* (5.8%, 18/311) (Figure 3).

### 3.3. Resistance by Antimicrobial Class Among Bacterial Species Between Cats and Dogs

**Figure 4:**
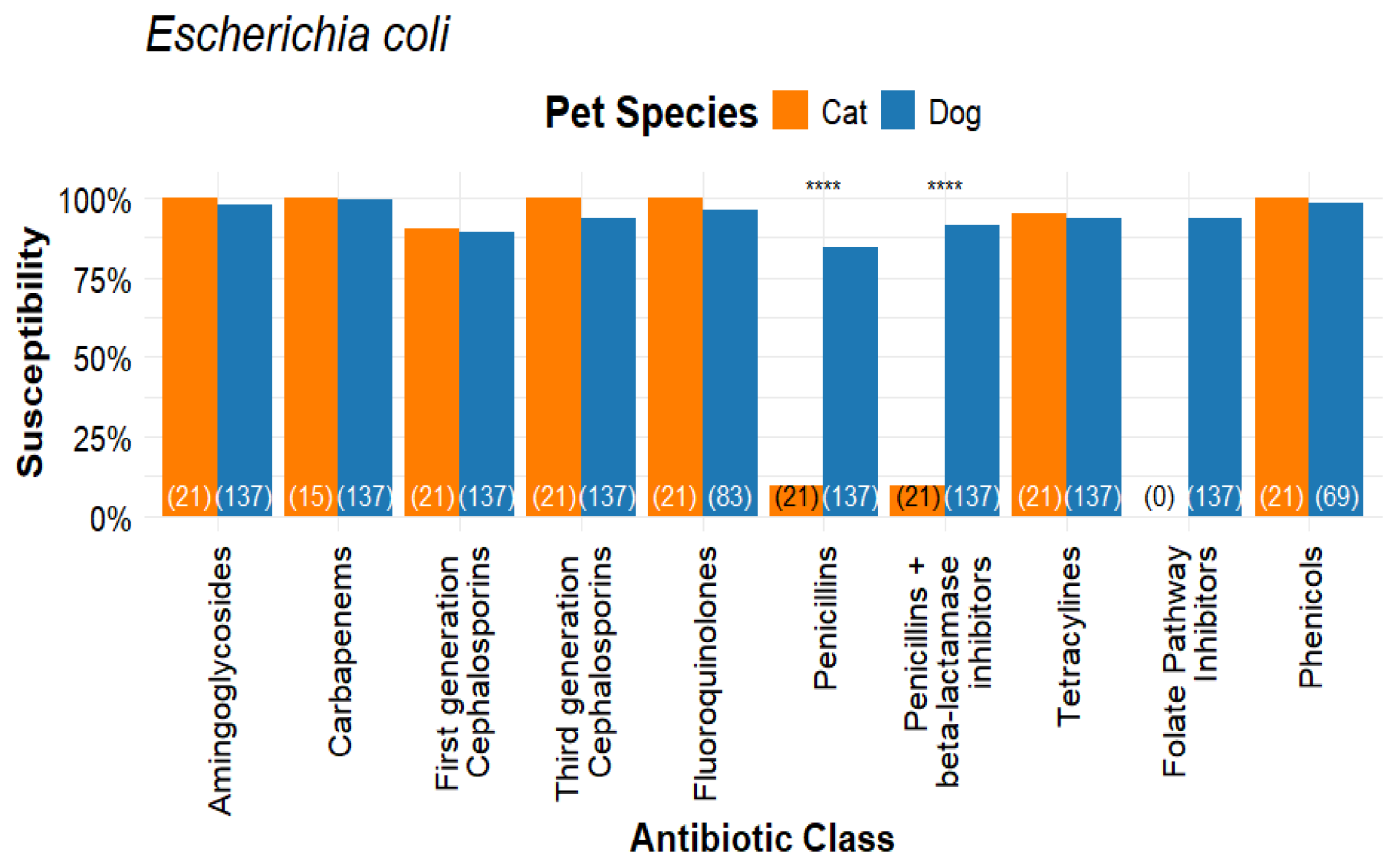
*Escherichia coli* isolates from cat and dog urine samples showing percentage of susceptibility to the tested classes of antibiotics. No antibiotic class results were excluded due to intrinsic resistance. Proportional differences between cats and dogs: **** = *p* < 0.0001. Numbers in bars represent total isolates tested for that antibiotic class.

**Figure 5:**
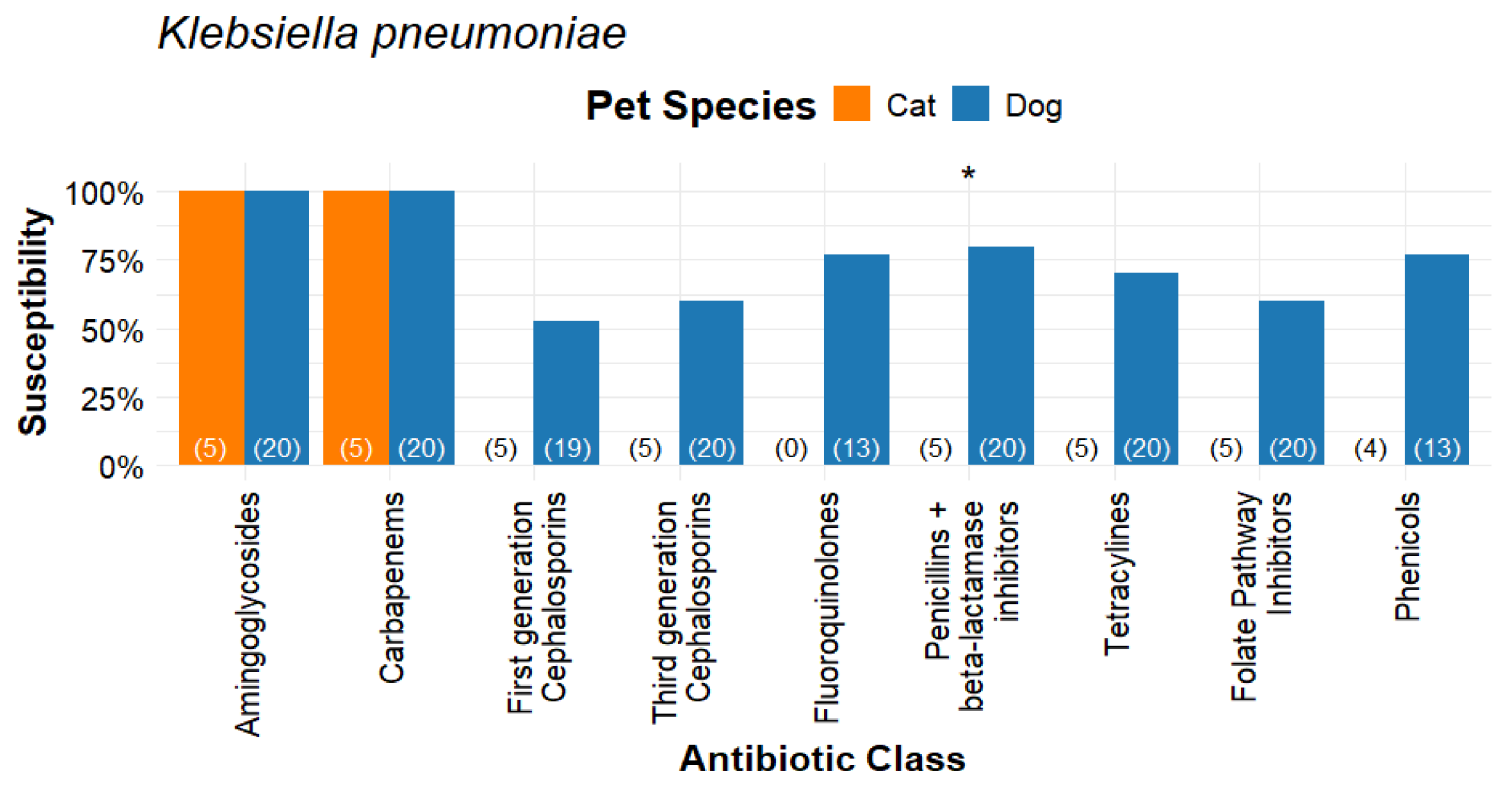
*Klebsiella pneumoniae* isolates from cat and dog urine samples showing percentage of susceptibility to the tested classes of antibiotics. The results for the penicillins (ampicillin), were excluded due to intrinsic resistance. The five *K. pneumoniae* isolates from cats produced no results for the Fluoroquinolones and 0% susceptibility (100% resistance) to the First and Third Generation Cephalosporins, Penicillin + Beta-Lactamase Inhibitors, and Folate Pathway Inhibitors. Proportional differences between cats and dogs: * = *p* < 0.05. Numbers in bars represent total isolates tested for that antibiotic class.

**Figure 6:**
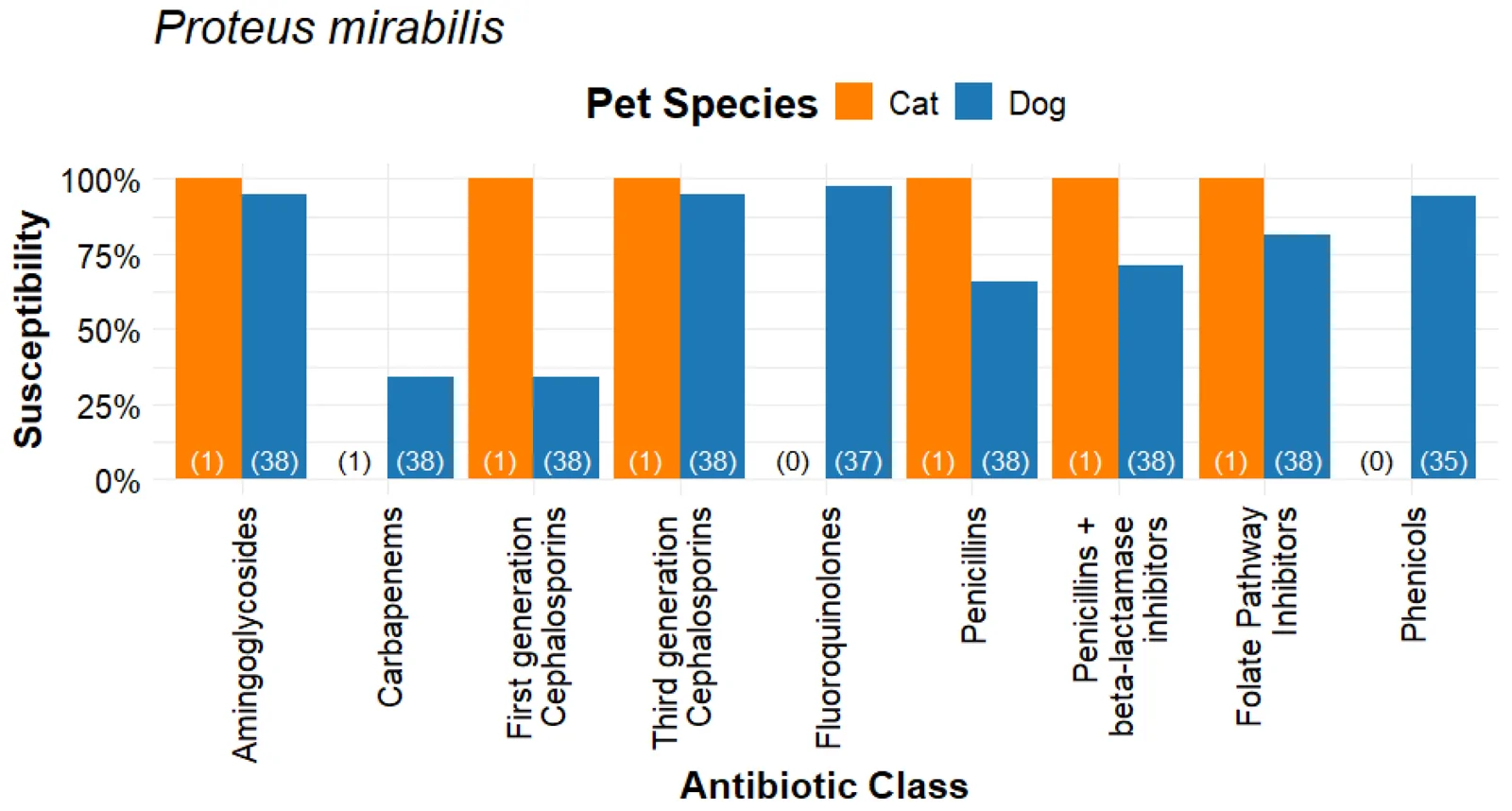
*Proteus mirabilis* isolates from cat and dog urine samples showing percentage of susceptibility to the tested classes of antibiotics. The results for the tetracyclines, doxycycline and tetracycline, were excluded due to intrinsic resistance. The one sample of *P. mirabilis* isolates from cats produced no results for the Fluoroquinolones and 0% susceptibility (100% resistance) to the Carbapenems. Numbers in bars represent total isolates tested for that antibiotic class.

Resistance by antimicrobial class among the 6 primary bacterial species between cats and dogs was evaluated by Chi-square with Yates correction and z-test for proportions with Benjamini-Hochberg adjustment for multiple comparisons. For the three main species of Gram-negative bacteria, the *Escherichia coli* isolates from cats demonstrated more resistance to penicillins (*p* = 1.94 × 10^−12^) and penicillins + beta-lactamase inhibitors (*p* = 5.9 × 10^−17^) when compared to *E. coli* isolates from dogs (Figure 4). *Klebsiella pneumoniae* cat isolates showed more resistance to penicillins + beta-lactamase inhibitors (*p* = 0.0203) when compared to samples from dogs (Figure 5). There was no difference in class resistance among all tested classes of antibiotics between feline and canine samples for *Proteus mirabilis* (Figure 6).

**Figure 7:**
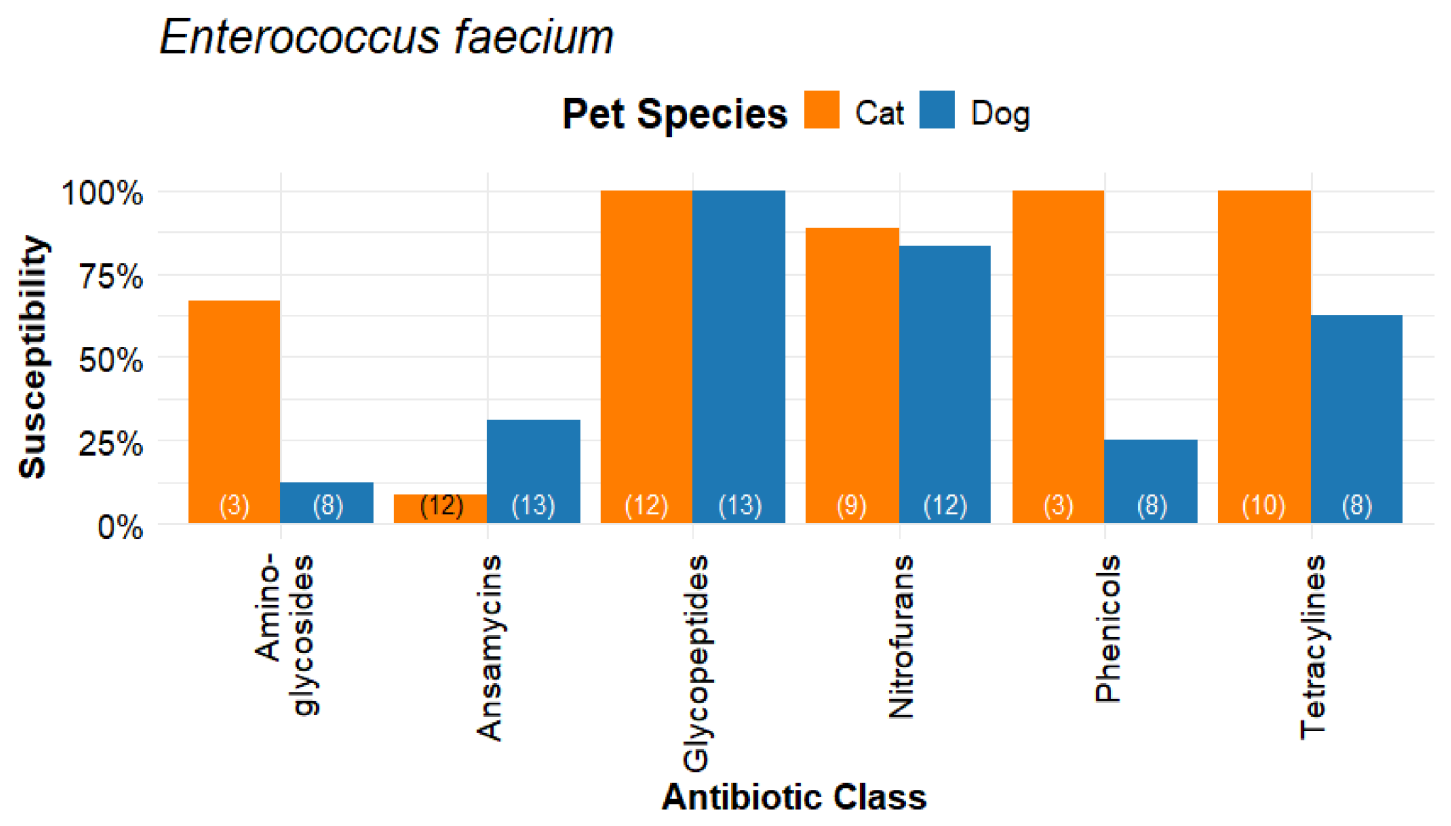
*Enterococcus faecium* isolates from cat and dog urine samples showing percentage of susceptibility to the tested classes of antibiotics. The results for the aminoglycosides, the cephalosporins, folate pathway inhibitors, macrolides, penicillins, penicillins + beta-lactamase inhibitors, and the carbapenem imipenem were excluded due to intrinsic resistance. Numbers in bars represent total isolates tested for that antibiotic class.

**Figure 8:**
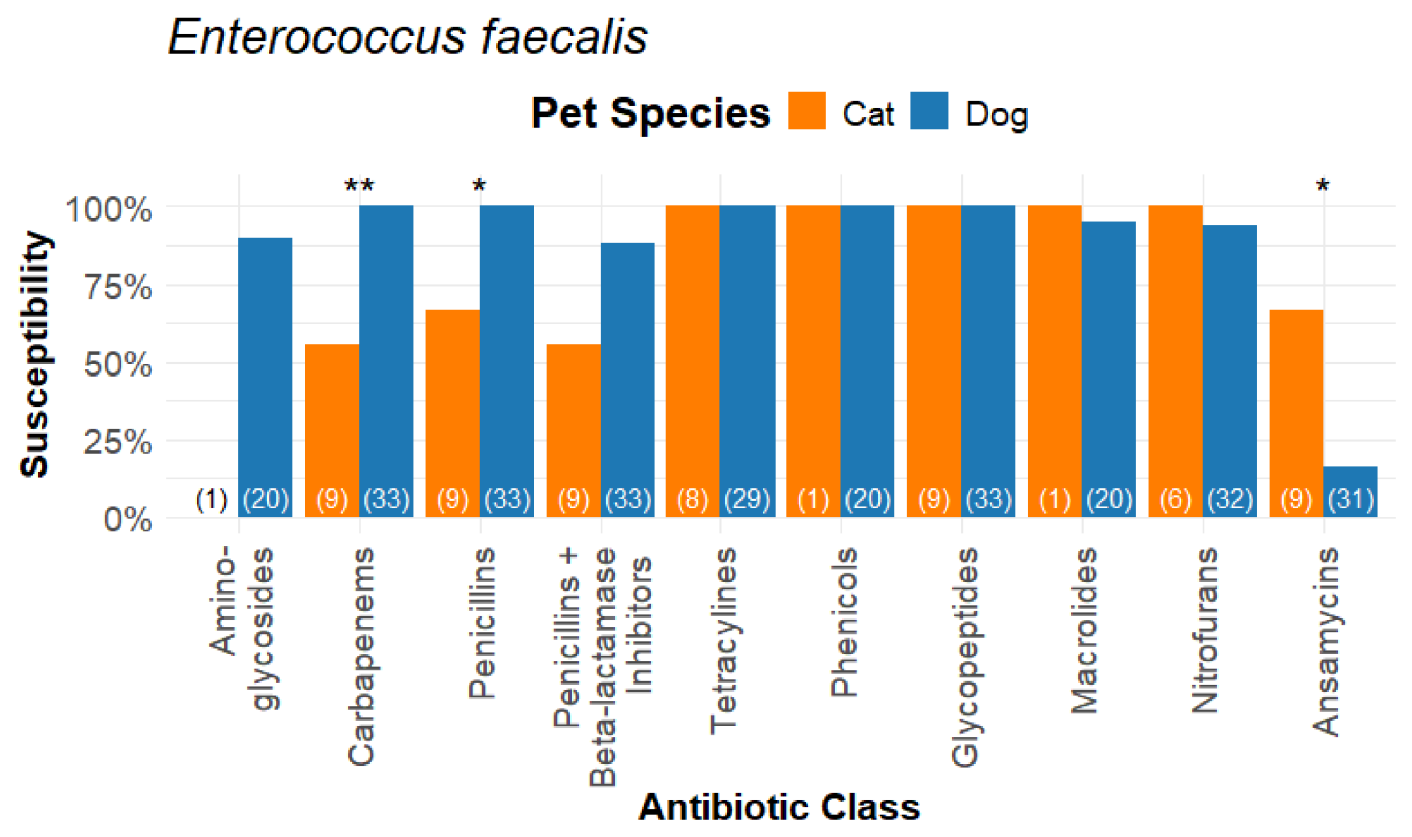
*Enterococcus faecalis* isolates from cat and dog urine samples showing percentage of susceptibility to the tested classes of antibiotics. The results for the folate pathway inhibitors and the first-generation cephalosporins were excluded due to intrinsic resistance. One sample of *E. faecalis* from cats produced 0% susceptibility (100% resistance) to the Aminoglycosides. Proportional differences between cats and dogs: * = *p* < 0.05; ** = *p* < 0.01. Numbers in bars represent total isolates tested for that antibiotic class.

**Figure 9:**
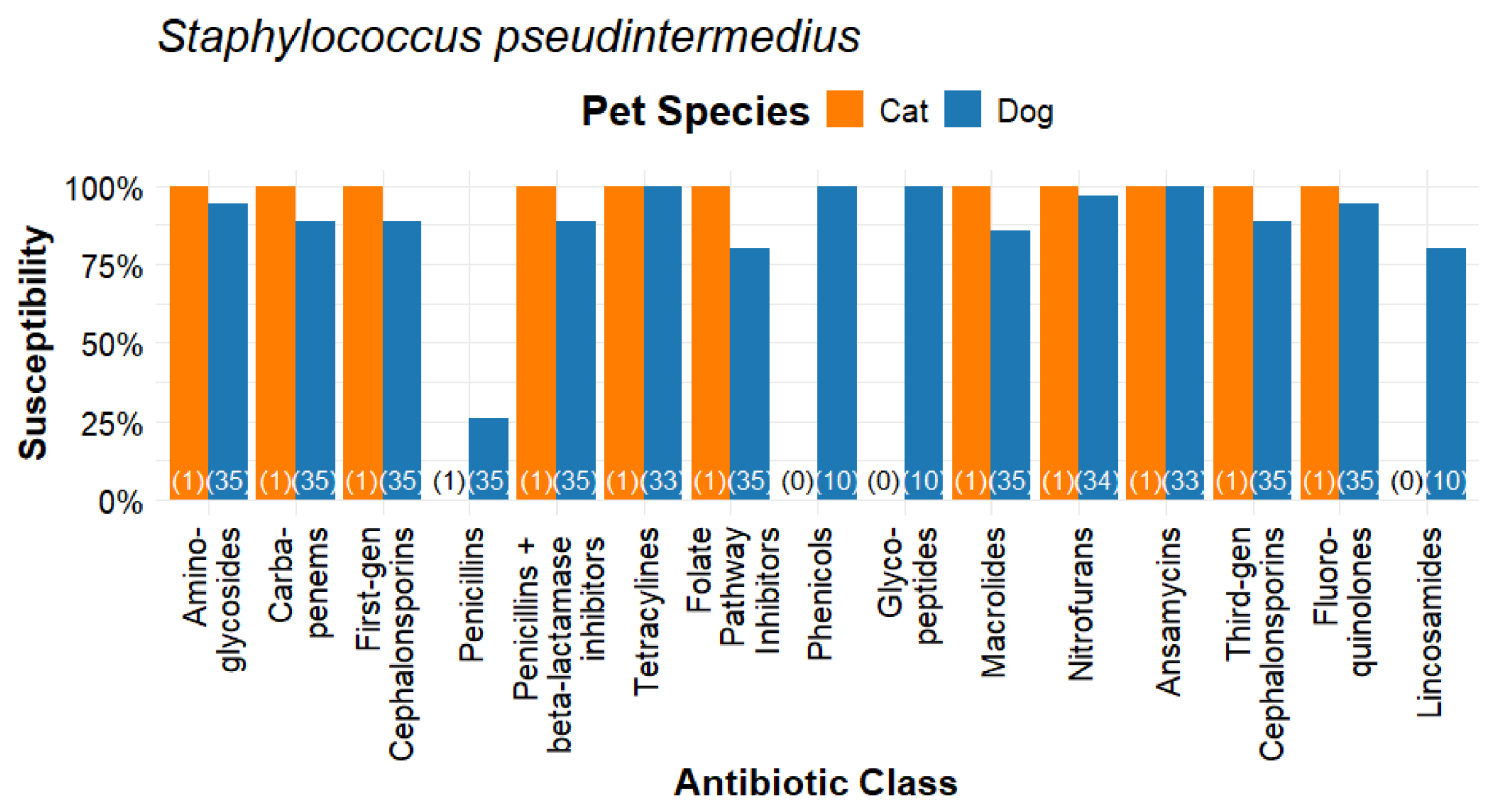
*Staphylococcus pseudintermedius* isolates from cat and dog urine samples showing percentage of susceptibility to the tested classes of antibiotics. No antibiotic class results were excluded due to intrinsic resistance. The one sample of *S. pseudintermedius* isolates from cats produced no results for the Phenicols, Glycopeptides, and Lincosamides with 0% susceptibility (100% resistance) to the Penicillins. Numbers in bars represent total isolates tested for that antibiotic class.

Among the three main species of Gram-positive bacteria, *Enterococcus faecium* resistance was comparable between cats and dogs (Figure 7). The *Enterococcus faecalis* isolates from cats, when compared to those from dogs, have significantly more resistance among the antimicrobial classes of carbapenems (adjusted *p* ≤ 0.001), penicillins (adjusted *p* ≤ 0.001), and penicillins + beta-lactamase inhibitors (*p* ≤ 0.05) (Figure 8). The results for *Staphylococcus pseudintermedius* isolates were comparable among all tested classes of antibiotics between cats and dogs (Figure 9).

### 3.4. Rate of Resistance to Antibiotic Agents for Gram-Negative and Gram-Positive Isolates

The rate of resistance for each tested antibiotic was calculated for the three main Gram-negative species of *Escherichia coli*, *Klebsiella pneumoniae,* and *Proteus mirabilis* as well as the three primary Gram-positive species of *Entercoccus faecalis*, *Enterococcus faecium*, and *Staphylococcus pseudintermedius* [35,36] (Tables S1–S4). The rates of resistance for the other 17 species (<5% of the total number of positive isolates) can be found in Tables S1 and S2.

The nitrofuran, nitrofurantoin, which is used in an extralabel fashion in dogs and cats to treat both Gram-positive and Gram-negative urinary tract infections, is included in the Sensititre^TM^ companion animal Gram-positive (COMPGP1F) plate and is not included in the companion animal Gram-negative (COMPGN1F) plate [43] (Table 1 and Tables S1–S4).

For the 158 isolates of *E. coli*, no antibiotics were excluded due to intrinsic resistance in the calculation of rate of resistance) [44] (Figure 4). The 21 feline *E. coli* isolates showed an extremely high rate of resistance to ampicillin (90.5%) as well as amoxicillin/clavulanic acid (90.9%). These feline samples showed low resistance to cefazolin (9.5%), trimethoprim/sulfamethoxazole (4.8%), and tetracycline (4.8%). A 0% rate of resistance was seen in the remaining 11 tested antibiotic agents, including three fluoroquinolones, enrofloxacin, marbofloxacin, and orbifloxacin, and the carbapenem imipenem (Tables S1 and S3) [45,46].

The 137 canine isolates of *E. coli* showed a moderate resistance (10.9%) to cefazolin. These isolates showed low resistance to ampicillin (8.8%), amoxicillin/clavulanic acid (8.8%), trimethoprim/sulfamethoxazole (6.6%), cefpodoxime (6.6%), and the two tetracyclines, tetracycline (6.6%) and doxycycline (5.8%). A low rate of 4.8% was recorded for cephalexin, 3.6% for both ceftazidime and gentamicin, and 1.5% for chloramphenicol. A very low rate of resistance was recorded for imipenem (0.7%), with a 0% rate of resistance seen for amikacin.

The penicillin class agent ampicillin was not included in either the feline or canine calculations for *Klebsiella pneumoniae* due to intrinsic resistance [47]. The five feline *K. pneumoniae* isolates demonstrated extremely high resistance to amoxicillin/clavulanic acid (90.5%) and 100% resistance to the first-generation cephalosporin cefazolin, trimethoprim/sulfamethoxazole, chloramphenicol, and doxycycline as well as the third-generation cephalosporins cefpodoxime and ceftazidime. These feline isolates also demonstrated a very high resistance (60%) to tetracycline with 0% resistance for the two aminoglycosides, amikacin and gentamicin, as well as the carbapenem, imipenem. No results were recorded for any of the fluoroquinolones used to test these feline isolates (Figure 5, Tables S1 and S3).

The 18 canine *K. pneumoniae* isolates showed 0% resistance to the aminoglycosides, gentamicin and amikacin, as well as to the carbapenem imipenem. The canine isolates produced high rates of resistance to the cephalosporins cefazoline (47.1%), cefpodoxime (44.4%), and ceftazidime (44.4%). High rates of resistance to trimethoprim/sulfamethoxazole (20.5%), chloramphenicol (23.1%), doxycycline (46.2%), tetracycline (46.2%), enrofloxacin (27.3%) and orbifloxacin (27.3%), were also recorded for these isolates. The *K. pneumoniae* canine isolates showed a moderate rate of resistance to amoxicillin/clavulanic acid (16.7%) and cephalexin (12.5%) with low resistance to marbofloxacin (9.1%) (Figure 5, Tables S1 and S3).

For the *Proteus mirabilis* isolates, the results for the tetracyclines, doxycycline and tetracycline, were excluded due to intrinsic resistance [48]. Except for 100% resistance to imipenem, the one feline sample showed a 0% rate of resistance or no measurable results to the 14 remaining antibiotics. For the 38 *P. mirabilis* canine isolates, a very high rate of resistance (65.8%) was seen for both cefazolin and imipenem. A high rate of resistance was recorded for ampicillin (34.2%), amoxicillin/clavulanic acid (23.7%), and cephalexin (35.1%). A moderate rate of resistance was seen in both piperacillin/tazobactam (10.5%) and trimethoprim/sulfamethoxazole (18.4%). Low rates of resistance were seen in isolates tested with cefpodoxime (2.6%), ceftazidime (2.6%), chloramphenicol (5.6%), and the three fluoroquinolones, enrofloxacin (2.7%), marbofloxacin (2.7%), and orbifloxacin (2.7%). The canine isolates tested for gentamicin produced a low rate of 5.3%, while only amikacin produced a resistance rate of 0% (Figure 6, Tables S1 and S3).

For *Enterococcus faecalis*, the results for both the folate pathway inhibitors and the first-generation cephalosporins were excluded in both feline and canine isolates due to intrinsic resistance [48] (Figure 7). The 9 feline *E. faecalis* isolates showed an extremely high resistance to gentamicin (100%). High resistance was recorded for amoxicillin/clavulanic acid (44.4%), imipenem (44.4%), both penicillins, ampicillin and penicillin (33.3%), and rifampin (33.3%). To complete the series of tested antibiotics, *E. faecalis* feline isolates showed 0% rates of resistance to chloramphenicol, the tetracyclines doxycycline, tetracycline, and minocycline, as well as erythromycin, nitrofurantoin, and vancomycin. The 33 *E. faecalis* canine isolates generated extremely high resistance rates to rifampin (83.8%) and a moderate resistance rate to amoxicillin/clavulanic acid (12.1%). Low rates of resistance to erythromycin (5.0%), nitrofurantoin (6.2%), and gentamicin (10%) were observed. The canine isolates produced a 0% rate of resistance to ampicillin, penicillin, imipenem, chloramphenicol, tetracycline, doxycycline, minocycline, and vancomycin (Tables S2 and S4).

*Enterococcus faecium*, which is intrinsically resistant to the aminoglycosides, the cephalosporins, folate pathway inhibitors, macrolides, penicillins, penicillins + beta-lactamase inhibitors, and the carbapenem imipenem, demonstrated the following rates of resistance to the remaining antibiotic classes [34] (Figure 8). In the 12 feline isolates, a 90% rate of resistance was observed for rifampin. A low rate of resistance was seen for nitrofurantoin (9.1%). For the remainder of tested antibiotic agents, vancomycin, tetracycline, minocycline, doxycycline, and chloramphenicol, the rate of resistance was 0%. The 13 canine isolates of *E. faecium* showed extremely high rates of resistance to chloramphenicol (85.7%). A high rate of resistance was recorded for doxycycline (25%), minocycline (37.5%), and tetracycline (37.5%) and a moderate rate of resistance for nitrofurantoin (15.4%). With the exception of vancomycin, none of these 13 canine isolates produced a 0% rate of resistance to any of the antibiotic agents tested (Tables S2 and S4).

For *Staphylococcus pseudintermedius*, none of the antibiotic classes were excluded due to intrinsic resistance [44] (Figure 9). The one feline sample produced 100% resistance to ampicillin and penicillin with 0% rates of resistance for the remainder of the antibiotic agents tested with recorded results. In contrast, the 35 canine isolates of *S. pseudintermedius* demonstrated extremely high rates of resistance to ampicillin (77.4%), penicillin (77.4%), and a moderate rate of resistance to oxacillin (11.4%). Moderate rates of resistance were also recorded for amoxicillin/clavulanic acid (11.4%), cefazolin (11.4%), cefovecin (11.4%), cephalothin (11.4%), cefpodoxime (11.4%), imipenem (11.4%), erythromycin (11.4%), trimethoprim/sulfamethoxazole (17.1%), and clindamycin (20%). In the canine isolates low rates of resistance were detected for gentamicin (5.7%), nitrofurantoin (2.9%), clindamycin (9.1%), enrofloxacin (5.7%), marbofloxacin (5.7%), and pradofloxacin (3.0%). Rates of 0% resistance were observed for rifampin, amikacin, chloramphenicol, doxycycline, minocycline, tetracycline, and vancomycin (Tables S2 and S4).

We investigated resistance trends for the six primary (≥5% of total positive isolates) bacteria over the six years covered in the study. Resistance remained stable in feline cases for *Klebsiella pneumoniae*, *Escherichia coli*, *Proteus mirabilis*, *Enterococcus faecium*, *Enterococcus faecalis,* and *Staphylococcus pseudintermedius*. In canines, *K. pneumoniae* showed a significant increase in tetracycline resistance, beginning in 2022 and continuing into 2023 (Cochran-Armitage trend test, adjusted *p*-value < 0.05). *E. faecalis* showed a significant decrease in resistance to penicillins-beta-lactamase inhibitors starting in 2018 (*p* < 0.05). *E. faecium* showed a significant increase in resistance to phenicols starting in 2021 (*p* < 0.05). *E. coli*, *P. mirabilis*, and *S. pseudintermedius* did not show any trends in resistance.

### 3.5. Determination of Multidrug Resistance (MDR)

The bacterial isolates that were resistant to one or more classes of the tested antibiotics were 54.9% (200/364) of the total isolates. The number of bacteria that produced resistance to four or more antibiotic classes made up 10.2% (37/364), 6.9% (27/364) were resistant to three classes, 13.2% (47/364) were resistant to two classes, and 24.7% (89/364) were resistant to one class. Bacterial isolates that demonstrated only intrinsic resistance totaled 9.6% (35/364) and 35.4% (129/364) were 0% resistant or 100% susceptible to the tested antibiotics. *Escherichia coli* (30.5%, 111/364) made up the largest group of 0% resistant or 100% susceptible bacteria. *Klebsiella pneumoniae* (39.1%, 9/23) and *Enterococcus faecalis* (83.3%, 10/12) were the major species for the Gram-negative and Gram-positive groups, respectively, for the only intrinsic resistance group of bacteria (Table 3).

Sixty-four (17.6%) of the 364 isolates were identified as multidrug-resistant (MDR). The Gram-negative isolates comprised 76.6% of the total MDR organisms, with *E. coli* (26.6%), *P. mirabilis* (28.1%), and *K. pneumoniae* (21.9%) being the majority of the 49 Gram-negative MDR isolates. Gram-positive bacteria made up 23.4% of the MDR isolates, with *E. faecalis* (9.4%) and *S. pseudintermedius* (10.9%) being the majority of the 15 Gram-positive bacteria. Four of the *S. pseudintermedius* isolates were phenotypically oxacillin resistant and were classified as methicillin-resistant *S. pseudintermedius* (MRSP) as well as MDR [34] (Table 4).

Specifically for the four canine phenotypically oxacillin-resistant *S. pseudintermedius* isolates, all were resistant to the tested penicillins, penicillin + beta-lactamase inhibitors, first-generation cephalosporins, third-generation cephalosporins, and the carbapenem, imipenem. Only two were resistant to the folate pathway inhibitor trimethoprim/sulfamethoxazole and the macrolide erythromycin. A single canine isolate was resistant to the aminoglycoside gentamicin.

There were a total of 53 canine and 11 feline MDR bacterial isolates. The primary bacteria contributing to these 53 canine MDR isolates at ≥5% of total canine MDRs were *Proteus mirabilis* (32.1%), *Escherichia coli* (28.3%), *Klebsiella pneumoniae* (17.0%), and *Staphylococcus pseudintermedius* (13.2%). For the 11 feline MDR isolates, *Enterococcus faecalis* (36.4%), *K. pneumoniae* (45.4%), and *E. coli* (18.2%) were the main contributing bacteria. (Table 4). The MDRs isolated from the other 17 species (<5% of the total number of positive isolates) can also be found in Table 3 and Table 4.

Because of the sparseness of the feline isolate data, we pooled the feline and canine data within bacterial species after first confirming that the proportion of MDR cases observed in *E. coli* in both species was similar (9.5% in feline versus 12.2% in canine isolates, *p* = 1), which is consistent with all animals originating from a single geographic region. Chi-square analysis of MDR occurrence followed by Fisher’s exact test of pairwise comparisons with Benjamini-Hochberg correction demonstrated that both *K. pneumoniae* (60.9%) and *P. mirabilis* (43.6%) had a higher proportion of MDR isolates than *E. coil* (10.8%), *E. faecalis* (14.3%), and *E. faecium* (4.0%); all adjusted *p*-values < 0.05). In addition, *K. pneumoniae* had a greater proportion of resistance compared to *S. pseudintermedius* (60.9% versus 19.4%, respectively, adjusted *p*-value < 0.05) (Table 3).

## 4. Discussion

Urinary tract infections are among the most common infections diagnosed in companion animals, and the available antimicrobials required for treatment are limited, with most considered critical to humans by the World Health Organization (WHO) [28]. These factors underlie the importance of a One Health perspective that includes the monitoring of antimicrobial resistance trends in the uropathogenic bacteria isolated from dogs and cats [18]. This study provides information on the occurrence of bacteria associated with feline and canine positive urine samples with the aim to better understand the antimicrobial resistance of pathogens isolated from 717 veterinary clinical cases in Arizona (USA), allowing us to describe the epidemiology of positive UTI samples and the antimicrobial resistance patterns of the bacterial etiologic agents over a six-year period.

Bacteriuria (≥1000 CFU/mL) was present in 42.7% of the cystocentesis-collected urine samples, and this value appears to be within the upper range of those reported in studies from the United Kingdom (29.9%), Portugal (30.4%), Spain (37%), the United States (46.2%), and Italy (52.6%) [37,49,50,51,52]. The UTI treatment guidelines published by the International Society for Companion Animal Infectious Diseases (ISCAID) recommended that the choice of the empirical drug should be based on the local frequency and predictable susceptibility patterns of bacterial pathogens [15]. We compared the overall susceptibility profiles of 364 isolates from 14 Gram-positive and 9 Gram-negative bacterial species. Our analysis showed that 64.6% of the isolates from this study were resistant to at least one antibiotic drug, highlighting the importance of correct identification and antimicrobial susceptibility testing.

Our study revealed that 48.9% (256/524) of dogs and 23.8% (48/193) of cats were positive for UTI bacterial pathogens, which was like other reports in cats and dogs [49,53,54]. Compared with previous studies, dogs accounted for 84.8% (256/302) of all positive samples in our study, a proportion that was 13.9–33.3 percentage points higher than previously reported. In contrast, cats represented 15.2% (46/302) of positive samples, which was 13.9–33.3 percentage points lower than values reported in other studies [35,37,52,55]. These differences were most likely due to the larger number of canine urine samples (524) and positive canine samples (256) compared with a smaller number of feline urine samples (193) and positive samples (46), a difference of 2.7× and 5.6×, respectively, identified in our study. Overall, this larger number of canine samples can be due to several factors: the larger number of dogs as patients and the diagnostic rate in dogs compared to cats [50,56,57].

In samples from female and male dogs, we observed a higher rate of UTI-related pathogens in females (81.2%) compared to males (18.8%), which is consistent with other studies [49,50,58]. In our work as well as previous studies, dogs and cats older than 6–11 years and older than 11 years were the most affected by UTI-related pathogens [37,50]. This can be due to an increased presence of predisposing factors in these age classes such as chronic kidney disease, endocrine disorders, urinary bladder distention, uroliths, urinary incontinence, and medications that may increase the risk of a urinary tract infection [37,52,58]. We found that the senior group (>6–11 years) played a role for only female and not male dogs. There was no association of positive urinary samples between age or sex in cats, which differs from recent results in studies by Dorsch et al. and Garcês et al. [25,37,59]. The lack of this age/sex association in cats could be due to the low number of positive feline samples in this study.

When using seasonal bins, a summer seasonal pattern for UTI-related pathogens was identified for both dogs and cats. A study by. Ojasanya et al. [60] also reported a summer increase in antibiotic susceptibility testing in cats and dogs. In comparison, Hernando et al. [54] discovered *Enterococcus* spp. were prevalent in summer, while *Escherichia* spp. and *Proteus* spp. were more frequently isolated in spring.

Among the Gram-negative and Gram-positive bacterial species, *Escherichia coli* was the most common bacterial isolate for UTI-related pathogens in both dogs (44.1%) and cats (39.6%), as seen in previous studies [50,52,53,54,55]. Canine *E. coli* isolates demonstrated very low to moderate rates of resistance to all antibiotics tested. Among the feline *E. coli* isolates, extremely high rates of resistance were recorded for both ampicillin and amoxicillin/clavulanic acid in cats. When a resistance prevalence has increased above the moderate rate of resistance threshold of 20%, patients should be withdrawn from these antibiotic therapies [53,61]. These findings suggest the use of a short-term course of trimethoprim/sulfamethoxazole for the treatment of feline *E. coli* UTIs diagnosed in the metropolitan area of Phoenix, AZ, USA.

Although *Klebsiella pneumoniae* ranked last among the primary bacterial UTI isolates in both cats (9.4%) and dogs (5.8%), *K. pneumoniae* isolates demonstrated high to extremely high resistance rates to the majority of first-line and second-line antibiotics recommended for the treatment of UTIs in companion animals. These *K. pneumoniae* resistance rates emphasize the importance of isolation and susceptibility testing of UTI isolates for definitive treatment with the possible use of analgesics prior to the initiation of antibiotic therapy in order to limit the selection for AMR *K. pneumoniae* bacteria by empiric therapy [62].

*Proteus mirabilis* ranked second among the canine isolates (12.2%) and unranked among the top feline bacterial isolates. Our findings support *P. mirabilis* as primarily a pathogen of canine UTIs, as seen in other studies [52,54,55]. There were no significant differences among the resistance of antibiotics used for testing in both dogs and cats. The canine isolates did reveal moderate-to-high resistance among the first-line treatments amoxicillin/clavulanic acid and trimethoprim/sulfamethoxazole. These results suggest possible second-line alternatives for the treatment of first-line resistant *P. mirabilis* UTIs for dogs in our study area.

Among the Gram-positive bacteria that made up 5% or more of the individual feline and canine UTI isolates, *Enterococcus faecalis* ranked fourth (10.6%) among canine isolates and third (17.0%) in feline UTI isolates. A similar ranking was found for dogs in a study completed in the United States [51]. No comparable study was identified for cats. In our study, *E. faecalis* urinary isolates were highly resistant to first- and second-line antibiotics, except for low resistance (0–6.2%) to nitrofurantoin in feline and canine isolates. A moderate resistance (12.1%) to amoxicillin/clavulanic acid was recorded among canine isolates. As possible local treatment options in Arizona veterinary practice, these observations suggest the use of nitrofurantoin when multidrug-resistant *E. faecalis* is involved in feline UTIs and amoxicillin/clavulanic acid as a possible first-line antibiotic for canines with *E. faecalis* positive urine samples.

*Enterococcus faecium* ranked second (22.6%) among feline isolates and was just below the cutoff of 5% at 4.2% among the canine isolates. Our canine findings appear to be closely aligned with a previous study in the USA [51]. The feline findings appear to vary from the normal expectation of 85% *E. faecalis* for cat isolates of *Enterococcus* species. In this study, *E. faecium* was 57.1%, while *E. faecalis* was 42.9% [59]. This variation could be due to geography or a low number of samples with *Enterococcus* species. The intrinsic multidrug resistance of *E. faecium* in both cats and dogs eliminates many of the first-line and second-line treatment options for urinary bacterial pathogens except for nitrofurantoin with low to moderate rates of resistance (9.1–15.4%). Based on these intrinsic multidrug-resistant results, nitrofurantoin appears to be an initial treatment option for both canine and feline MDR *E. faecium*-positive urine samples in our study area [15].

From the thirty-six isolates of *Staphylococcus pseudintermedius* isolates, only one sample was identified from cats, while thirty-five were isolated from canine urine samples. This predominance of *S. pseudintermedius* from canines was also seen in a study from Italy [52]. For antimicrobial resistance, there was no significant difference between the feline and canine *S. pseudintermedius* isolates. This result differs from studies in Germany and Spain, where feline *S. pseudintermedius* isolates were significantly more resistant [49,53]. This lack of significance in our study was most likely due to the single feline isolate among the 36 samples tested. Both the single feline and thirty-five canine isolates demonstrated extremely high rates of resistance (75–100%) to the penicillins, ampicillin and penicillin, with moderate resistance to oxacillin (11.4%). The latter was due to four canine phenotypic oxacillin-resistant samples. In general, these *S. pseudintermedius* isolates were susceptible to the first-line antibiotics of amoxicillin/clavulanic acid and trimethoprim/sulfamethoxazole as well as the second-line third-generation cephalosporins, nitrofurantoin, and fluroquinolones. Due to the presence of oxacillin resistance and the approximate 20% resistance rate to trimethoprim/sulfamethoxazole observed in dogs, we suggest that Arizona veterinarians carefully monitor the effectiveness of empiric first-line antibiotics when treating S. *pseudintermedius* urinary tract infections in both dogs and cats.

The Gram-negative multidrug-resistant feline *Escherichia coli* isolates composed 18.2% of the samples and are in the broad range (2.75–22.5%) of four other studies [35,49,52,53]. The frequency of MDR *E. coli* canine isolates (28.3%) was higher than observed in these same studies (3.4–22.4%). The frequency of multidrug resistant *Klebsiella pneumoniae* isolates in cats and dogs, 45.4% and 17.0%, respectively, was lower than reported in Italy (100% and 45%) but higher than in Spain (3.5% and 2.5%). In Germany, MDR *K. pneumoniae* isolate percentages were lower in cats (25%) and higher in dogs (21.1%) [49,52,53]. The percentage (32.1%) of canine *P. mirabilis* MDR isolates was within the broad range (9.5–84.8%) of these same three studies as well as a study of MDRs from multiple European clinics [35].

For the MDR Gram-positive isolates, the percentage of *Enterococcus faecalis* feline isolates (36.4%) is like the findings of two other studies, while the frequency of canine isolates (3.8%) was dramatically less than the 44.8–50% found in these same studies [52,53]. *Enterococcus faecium* canine isolates produced a much lower percentage (1.9%) than those in an Italian study (81.9%), while there were no feline *E. faecium* MDR isolates to compare with other studies [49,52]. The feline *Staphylococcus pseudintermedius* MDR isolates (0%) were below the range of cases (4.4–66.6%) identified in studies from Italy and Spain [49,52]. The 13.2% of *S. pseudintermedius* MDR canine isolates was within the frequency range (2.4–75%) of four other studies [35,49,52,53].

A major aspect of these six specific genera of MDR bacteria is their presumed zoonotic or reverse-zoonotic potential. Each of these six genera has the potential for transfer from companion animals to humans or from humans to companion animals based on the identification of the same serotypes, resistant determinants, and plasmid profiles [63]. Specifically, multiple risks exist between humans and dogs due to close cohabitation and physical contact such as petting, licking, and shared sleeping spaces [63,64,65]. In general, this emergence and potential spread of AMR through both MDR and non-MDR genera, especially in canines and felines, poses a substantial One Health challenge, given the close interdependence of human, animal, and environmental health [66].

The limitations of this study include the small number of isolates and the localized distribution of samples within the state of Arizona. In the analysis of resistance rates, the grouping of intermediate isolates with susceptible isolates makes our estimates conservative. In addition, evaluation of a more significant number of isolates from other surrounding regions within the United States may be helpful to better investigate the antibiotic resistance patterns and examine whether a specific resistance profile is associated with the Arizona region. Not all clinical data regarding breed, history of complicated, relapsing, or recurrent UTIs, systemic comorbidities, or previous antibiotic treatments were available. Furthermore, previous antibiotic treatments could have affected antibiotic resistance profiles, or the resistance levels of the bacterial species may be overvalued due to the number of bacteria isolated from potential recurrent UTI samples.

## 5. Conclusions

This first study performed in Arizona, USA, provides important insights into the epidemiology of UTI pathogens and provides susceptibility patterns for the treatment of urinary tract infections in companion animals in the Phoenix metropolitan area of the state. These results emphasize the importance of bacterial culture and antimicrobial susceptibility testing before the initiation of antibiotic treatment to limit the selection for antibiotic-resistant isolates through prolonged ineffective treatment of urinary tract infections in dogs and cats. Finally, this information will contribute to the role antimicrobial resistance surveillance plays in the prevention and control of UTI pathogens in companion animals. Because dogs and cats often are in close contact with their owners, they may be potential sources for human infection and a One Health concern.

## Figures and Tables

**Table 1:** The antibiotic classes, agents, and concentrations used in (a) the companion animal Gram-negative (COMPGN1F) and in (b) the Gram-positive (COMPGP1F) 96-well plates for antimicrobial susceptibility tests.

(a) Companion animal plate COMPGN1F for Gram-negative bacteria antimicrobial susceptibility tests.		
**Antibiotic Classes**	**Antibiotic Agents**	**Dilution Range (μg/mL)**
Aminoglycosides	Amikacin	4.0–8.0
	Gentamicin	0.25–8.0
Carbapenems	Imipenem	1.0–8.0
Cephalosporins 1st Generation	Cefazolin	1.0–32.0
	Cephalexin	0.5–16.0
Cephalosporins 3rd Generation	Cefovecin	0.25–8.0
	Cefpodoxime	1.0–8.0
	Ceftazidime	4.0–16.0
Fluoroquinolones	Enrofloxacin	0.12–4.0
	Marbofloxacin	0.12–4.0
	Orbifloxacin	1.0–8.0
	Pradofloxacin	0.25–2.0
Folate Pathway Inhibitors	Trimethoprim/sulfamethoxazole	0.5/9.5–4.0/76.0
Penicillins	Ampicillin	0.25–8.0
Penicillins + Beta-lactamase Inhibitors	Amoxicillin/Clavulanic Acid 2:1 Ratio	0.25/0.12–8.0/4.0
	Piperacillin/Tazobactam constant 4	8.0/4.0–64.0/4.0
Phenicols	Chloramphenicol	2.0–32.0
Tetracyclines	Doxycycline	0.25–8.0
	Tetracycline	4.0–16.0
(b) Companion animal plate COMPGP1F for Gram-positive bacteria antimicrobial susceptibility tests.		
**Antibiotic Classes**	**Antibiotic Agents**	**Dilution Range (μg/mL)**
Aminoglycosides	Amikacin	16.0–32.0
	Gentamicin	4.0–16.0
Ansamycins	Rifampin	1.0–2.0
Carbapenems	Imipenem	1.0–4.0
Cephalosporins 1st Generation	Cefazolin	2.0–4.0
	Cephalothin	2.0–4.0
Cephalosporins 3rd Generation	Cefovecin	0.06–8.0
	Cefpodoxime	2.0–8.0
Fluoroquinolones	Enrofloxacin	0.25–4.0
	Marbofloxacin	1.0–4.0
	Pradofloxacin	0.25–2.0
Folate Pathway Inhibitors	Trimethoprim/sulfamethoxazole	2.0/38.0–4.0/76.0
Lincosamides	Clindamycin	0.5–4.0
Macrolides	Erythromycin	0.25–4.0
Nitrofurans	Nitrofurantoin	16.0–64.0
Penicillins	Ampicillin	0.25–8.0
	Oxacillin + 2% NaCl	0.25–2.0
	Penicillin	0.06–8.0
Penicillins + Beta-lactamase Inhibitors	Amoxicillin/Clavulanic Acid 2:1 Ratio	0.25/0.12–8.0/4.0
Phenicols	Chloramphenicol	8.0–32.0
Tetracyclines	Doxycycline	0.12–0.5
	Minocycline	0.5–2.0
	Tetracycline	0.25–1.0
Glycopeptides	Vancomycin	1.0–16.0

**Table 2:** The total and yearly frequencies as well as numbers for each species of bacteria isolated from positive cultures of urine samples from dogs and cats submitted to the Veterinary Microbiology Laboratory from 2018 to 2023.

**Frequency (%) for Dogs (N, 311)**							
**Bacterial Species**	**2018** **(N, 34)**	**2019** **(N, 57)**	**2020** **(N, 73)**	**2021** **(N, 38)**	**2022** **(N, 59)**	**2023** **(N, 50)**	**2018–2023** **(N, 311)**
*Escherichia coli*	29.4 (10/34)	43.8 (25/57)	38.4 (28/73)	39.5 (15/38)	44.1 (26/59)	66 (33/50)	44.1 (137/311)
*Staphylococcus pseudintermedius*	14.7 (5/34)	15.8 (9/57)	6.8 (5/73)	21.1 (8/38)	8.5 (5/59)	6 (3/50)	11.2 (35/311)
*Staphylococcus aureus*	0	1.8 (1/57)	0	0	0	0	0.3 (1/311)
*Staphylococcus intermedius*	0	3.5 (2/57)	0	0	0	0	0.6 (2/311)
*Enterococcus faecalis*	8.8 (3/34)	8.8 (5/57)	15.1 (11/73)	7.9 (3/38)	11.9 (7/59)	8 (4/50)	10.6 (33/311)
*Enterococcus faecium*	5.9 (2/34)	3.5 (2/57)	8.2 (6/73)	2.6 (1/38)	3.4 (2/59)	0	4.2 (13/311)
*Enterococcus gallinarum*	0	1.8 (1/57)	0	0	1.7 (1/59)	0	0.6 (2/311)
*Enterococcus canintestini*	0	1.8 (1/57)	0	0	0	0	0.3 (1/311)
*Proteus mirabilis*	5.9 (2/34)	12.3 (7/57)	16.4 (12/73)	13.2 (5/38)	15.2 (9/59)	6 (3/50)	12.2 (38/311)
*Klebsiella pneumoniae*	5.9 (2/34)	1.8 (1/57)	6.8 (5/73)	5.3 (2/38)	6.84 (4/59)	8 (4/50)	5.8 (18/311)
*Klebsiella variicola*	0	0	0	2.6 (1/38)	0	0	0.3 (1/311)
*Klebsiella oxytoca*	0	0	0	2.6 (1/38)	0	0	0.3 (1/311)
*Streptococcus canis*	2.9 (1/34)	1.8 (1/57)	2.7 (2/73)	2.6 (1/38)	1.7 (1/59)	4 (2/50)	2.6 (8/311)
*Streptococcus gallolyticus*	0	1.8 (1/57)	0	0	0	0	0.3 (1/311)
*Corynebacterium urealyticum*	5.9 (2/34)	0	0	0	0	0	0.6 (2/311)
*Actinomyces canis*	5.9 (2/34)	0	0	0	0	0	0.6 (2/311)
*Enterobacter cloacae*	2.9 (1/34)	0	2.7 (2/73)	0	3.4 (2/59)	2 (1/50)	1.9 (6/311)
*Pasteurella canis*	5.9 (2/34)	0	0	0	1.7 (1/59)	0	1.0 (3/311)
*Pseudomonas aeruginosa*	2.9 (1/34)	1.8 (1/57)	2.7 (2/73)	2.6 (1/38)	1.7 (1/59)	0	1.9 (6/311)
*Providencia stuartii*	2.9 (1/34)	0	0	0	0	0	0.3 (1/311)
**Frequency (%) for Cats (N, 53)**							
**Bacterial Species**	**2018** **(N, 2)**	**2019** **(N, 20)**	**2020** **(N, 21)**	**2021** **(N, 10)**	**2022** **(N, 0)**	**2023** **(N, 0)**	**2018–2023** **(N, 53)**
*Escherichia coli*	50 (1/2)	25 (5/20)	42.8 (9/21)	60 (6/10)	0	0	39.6 (21/53)
*Staphylococcus pseudintermedius*	0	0	4.8 (1/21)	0	0	0	1.9 (1/53)
*Staphylococcus felis*	0	0	4.8(1/21)	0	0	0	1.9 (1/53)
*Enterococcus faecalis*	50 (1/2)	20 (4/20)	9.5 (2/21)	20 (2/10)	0	0	17.0 (9/53)
*Enterococcus faecium*	0	35 (7/20)	23.8 (5/21)	0	0	0	22.6 (12/53)
*Proteus mirabilis*	0	0	0	10 (1/10)	0	0	1.9 (1/53)
*Klebsiella pneumoniae*	0	15 (3/20)	9.5 (2/21)	0	0	0	9.4 (5/53)
*Streptococcus gallolyticus*	0	0	4.8 (1/21)	0	0	0	1.9 (1/53)
*Corynebacterium auriscanis*		0	0	10 (1/10)	0	0	1.9 (1/53)
*Lactobacillus acidophilus*	0	5 (1/20)	0	0	0	0	1.9 (1/53)

**Table 3:** The Gram-negative and Gram-positive bacteria isolated from positive urine samples identified by number per group of intrinsic resistance, susceptibility, resistance of each species to classes of antibiotics, and the percent MDRs per bacterial species. MDR bacteria are a total of isolates resistant to both the three and four or more classes of antibiotics.

Bacteria	Only Intrinsic Resistance	100% Susceptibility	1 Class	2 Classes	3 Classes	≥4 Classes	Total MDR	Total Number Bacteria	% Species MDR
**Gram-negative Species**									
*Escherichia coli*	0	111	6	24	9	8	17	158	10.8
*Enterobacter cloacae*	4	0	0	1	1	0	1	6	16.7
*Klebsiella pneumoniae*	9	0	0	0	1	13	14	23	60.9
*Klebsiella oxytoca*	0	0	1	0	0	0	0	1	0
*Klebsiella variicola*	0	0	1	0	0	0	0	1	0
*Pasteurella canis*	0	3	0	0	0	0	0	3	0
*Proteus mirabilis*	4	0	14	4	8	9	17	39	43.6
*Providencia stuartii*	0	0	1	0	0	0	0	1	0
*Pseudomonas aeruginosa*	6	0	0	0	0	0	0	6	0
**Totals** **Gram-negative**	23	114	23	29	19	30	49	238	
**Gram-positive Species**									
*Actinomyces canis*	0	2	0	0	0	0	0	2	0
*Corynebacterium auriscanis*	0	0	1	0	0	0	0	1	0
*Corynebacterium urealyticum*	0	0	1	0	0	1	1	2	50.0
*Enterococcus canintestini*	0	0	1	0	0	0	0	1	0
*Enterococcus faecalis*	10	0	21	5	5	1	6	42	14.3
*Enterococcus faecium*	1	0	17	6	1	0	1	25	4.0
*Enterococcus gallinarum*	1	0	1	0	0	0	0	2	0
*Lactobacillus acidophilus*	0	0	1	0	0	0	0	1	0
*Staphylococcus aureus*	0	0	1	0	0	0	0	1	0
*Staphylococcus felis*	0	1	0	0	0	0	0	1	0
*Staphylococcus intermedius*	0	1	1	0	0	0	0	2	0
*Staphylococcus pseudintermedius*	0	8	18	3	2	5	7	36	19.4
*Streptococcus canis*	0	2	3	3	0	0	0	8	0
*Streptococcus gallolyticus*	0	1	0	1	0	0	0	2	0
**Totals** **Gram-positive**	12	15	66	18	8	7	15	126	
**Grand Totals**	35	129	89	47	27	37	64	364	
**Percent Totals**	9.6	35.4	24.7	13.2	6.9	10.2	17.3	100	

The six primary species of bacteria (≥5% of total isolated Gram-positive and Gram-negative bacteria) are highlighted. Bold letters are used for column headings, to identify the groups Gram-negative and Gram-positive species of bacteria, and totals as well as grand totals of specific groups of bacteria.

**Table 4:** Numbers and percentages of multidrug resistant bacteria by species isolated from cats and dogs, with the total and percent total of the multidrug resistant isolates from both cats and dogs.

**Gram-Negative Bacteria**	**CAT MDRs** **(% Cat Total 11)**	**DOG MDRs** **(% Dog Total 53)**	**Total (% Total) MDRs**
*Escherichia coli*	2 (18.2)	15 (28.3)	17 (26.6)
*Enterobacter cloacae*	0	1 (1.9)	1 (1.6)
*Klebsiella pneumoniae*	5 (45.4)	9 (17.0)	14 (21.9)
*Klebsiella oxytoca*	0	0	0
*Klebsiella variicola*	0	0	0
*Pasteurella canis*	0	0	0
*Proteus mirabilis*	0	17 (32.1)	17 (28.1)
*Providencia stuartii*	0	0	0
*Pseudomonas aeruginosa*	0	0	0
**Gram-negative** **Totals (Percent)**	7 (63.6))	42 (79.2)	49 (76.6)
**Gram-positive Bacteria**	**CAT MDRs** **(% Cat Total 11)**	**DOG MDRs** **(% Dog Total 53)**	**Total (% Total) MDRs**
*Actinomyces canis*	0	0	0
*Corynebacterium auriscanis*	0	0	0
*Corynebacterium urealyticum*	0	1 (1.9)	1 (1.6)
*Enterococcus canintestini*	0	0	0
*Enterococcus faecalis*	4 (36.4)	2 (3.8)	6 (9.4)
*Enterococcus faecium*	0	1 (1.9)	1 (1.6)
*Enterococcus gallinarum*	0	0	0
*Lactobacillus acidophilus*	0	0	0
*Staphylococcus aureus*	0	0	0
*Staphylococcus felis*	0	0	0
*Staphylococcus intermedius*	0	0	0
*Staphylococcus pseudintermedius*	0	7 (13.2)	7 (10.9)
*Streptococcus canis*	0	0	0
*Streptococcus gallolyticus*	0	0	0
**Gram-positive** **Totals (Percent)**	4 (36.4)	11 (20.8)	15 (23.4)
**Grand Totals** **(Percent Total 64 MDRs)**	11 (17.2)	53 (82.8)	64 (100)

The six primary species of bacteria (≥5% of total isolated Gram-positive and Gram-negative bacteria) are highlighted. Bold letters are used for column headings, to identify the groups of Gram-negative and Gram-positive species of bacteria, and totals as well as grand totals of MDR bacteria.

## Data Availability

The raw data supporting the conclusions of this article will be made available by the authors on request.

## Supplementary Materials

The following supporting information can be downloaded at: https://www.mdpi.com/article/10.3390/microorganisms14081655/s1. Table S1: Total numbers and rates of resistance to each tested antibiotic agent for Gram-negative bacteria isolated from positive dog and cat urine samples. The total number of dog and cat isolates for each bacterial species can be found in the left column. In the table, X represents no isolates tested, NR for no results for tested isolates, and INTRINSIC for intrinsic resistance of a tested species of bacteria.; Table S2: Total numbers and rates of resistance to each tested antibiotic agent for Gram-positive bacteria isolated from positive dog and cat urine samples. The total number of dog and cat isolates for each bacterial species can be found in the left column. In the table, X represents no isolates tested, NR for no results for tested isolates, and INTRINSIC for intrinsic resistance of a tested species of bacteria.; Table S3: The level and rates of resistance to each tested antibiotic agent for the three primary Gram-negative bacteria isolated from positive dog and cat urine samples. The number of dog and cat isolates for each bacterial species can be found at the top of the columns with X representing no isolates tested, NR for no results for tested isolates, and the intrinsic resistance to an antibiotic class highlighted in plum.; Table S4: The level and rates of resistance to each tested antibiotic agent for the three primary Gram-positive bacteria isolated from positive dog and cat urine samples. The number of dog and cat isolates for each bacterial species can be found at the top of the columns with X representing no isolates tested, NR for no results for tested isolates, and the intrinsic resistance to an antibiotic class highlighted in plum.
